# Propensity score matching analysis of the prognosis for the rare insular subtype of thyroid cancer based on SEER database

**DOI:** 10.18632/oncotarget.21826

**Published:** 2017-10-11

**Authors:** Yan Liu, Zeming Liu, Qiuyang Zhao, Teng Hua, Shuqi Chi, Tao Huang, Hongbo Wang

**Affiliations:** ^1^ Department of Obstetrics and Gynecology, Union Hospital, Tongji Medical College, Huazhong University of Science and Technology, Wuhan 430022, China; ^2^ Department of Breast and Thyroid Surgery, Union Hospital, Tongji Medical College, Huazhong University of Science and Technology, Wuhan 430022, China

**Keywords:** insular thyroid carcinoma, thyroid cancer, prognosis, SEER, propensity score matching

## Abstract

Insular thyroid carcinoma (ITC) is an uncommon thyroid malignancy with an unclear prognosis. The aim of this study was to determine the prognoses of patients with ITC. We investigated a large cohort of patients with differentiated thyroid cancer from the Surveillance, Epidemiology, and End Results (SEER) database who were registered between 2004 and 2013, and compared the prognosis of patients with ITC to those with classic papillary thyroid cancer (CPTC) and follicular thyroid cancer (FTC). Patient mortality was determined using Kaplan-Meier analyses with log-rank tests, as well as Cox proportional hazards regression analyses. The study cohort comprised of 165 patients with ITC, 5419 patients with FTC, and 60739 patients with CPTC. The rate of cancer-specific mortality per 1000 person-years for ITC was higher than that for CPTC or FTC. According to multivariate Cox regression analysis, however, the cancer-specific and all-cause mortality rates of ITC were similar to those of CPTC and FTC. The cancer-specific survival rate in patients with ITC was higher than that in patients with CPTC, but similar to that in patients with FTC, after adjusting for potentially influencing factors using propensity score matching analysis. These findings, which contrast with previously published data, provide new implications for the treatment of patients with ITC.

## INTRODUCTION

Thyroid cancer is the most common type of endocrine malignancy, and its incidence has been rising rapidly in recent decades [1–4]. The majority of these malignancies are differentiated thyroid cancers (DTC), of which papillary thyroid cancer (PTC) accounts for approximately 85% [5]. There are some rare variants of PTC, such as the tall cell, columnar cell, and diffuse sclerosing variants, as well as insular thyroid carcinoma (ITC) and Hürthle cell (oncocytic, oxyphilic) carcinoma [6–10].

ITC is a rare thyroid malignancy that was first defined by Carcangiu et al. in 1984 [11]. Its morphological features lie between those of well-differentiated carcinoma (papillary or follicular) and undifferentiated or anaplastic carcinoma of the thyroid [12, 13]. This type of thyroid carcinoma is considered an aggressive cancer with a high propensity for local recurrence and distant metastasis [14, 15]; it reportedly has a worse prognosis than classic thyroid carcinomas [9, 12, 13, 15]. However, some studies did not find a significant difference in prognosis between patients with ITC and those with differentiated thyroid cancer: papillary and/or follicular thyroid carcinomas (FTCs) [16, 17]. Additionally, most of the ITC-related literature comprises of case reports or small single institution-based case series, with no large population-level studies.

The Surveillance, Epidemiology, and End Results (SEER) program of the National Cancer Institute is the largest publicly available database of its kind, and is a valued source of high-quality information for cancer incidence and survival in the United States [18, 19]. Propensity score matching (PSM) is a statistical normalization method for analyzing observational data by estimating the effects of a treatment, policy, or other intervention and accounting for covariates that influence the administration of the treatment. The propensity score is a balancing score: The differences between groups on the covariates condensed down into a single score so if two groups balanced on the propensity score then balanced on all the covariates. PSM aims to reduce bias due to confounding variables [20]. In this study, we investigated the prognosis of ITC compared to PTC and FTC by analyzing the SEER database information from 2004–2013 using PSM methods due to the diversity of number of cases with different subtypes.

## RESULTS

### Demographic and clinical features

A total of 66323 patients with different histological subtypes (ITC, *n* = 165; classic papillary thyroid cancer [CPTC], *n* = 60739; and FTC, *n* = 5419) were included in this study. Patients received follow-up until December 2013. The study patients’ mean ages and follow-up durations according to their different histological subtypes are shown in Table 1.

**Table 1:** Characteristics for patients with different histological types

Covariate	level	Histological types		
		ITC	CPTC (*n* = 60739)	FTC (*n* = 5419)
		(*n* =165)		
Age		61.39 ± 16.43	48.37 ± 15.36	50.79 ± 17.29
Sex	Female	85 (51.5%)	46786 (77.1%)	3843 (70.9%)
	Male	80 (48.5%)	13953 (22.9%)	1576 (29.1%)
Race	White	131 (79.9%)	49651 (82.8%)	4186 (78.3%)
	Black	16 (9.8%)	3159 (5.3%)	640 (12.0%)
	Other	17 (10.3%)	7133 (11.9%)	517 (9.7%)
T stage	T1	6 (3.8%)	37974 (63.8%)	1240 (23.7%)
	T2	27 (17.0%)	8062 (13.6%)	2110 (40.4%)
	T3	80 (50.3%)	10845 (18.2 %)	1682 (32.2%)
	T4	46 (28.9%)	2599 (4.4%)	191 (3.7 %)
N-stage	N0	117 (76.0%)	44102 (74.9 %)	5114 (96.9%)
	N1	37 (24.0%)	14744 (25.1%)	161 (3.1 %)
M-stage	M0	128 (77.6 %)	59951 (98.7%)	5093 (94.0 %)
	M1	37 (22.4%)	788 (1.3 %)	326 (6.0%)
Multifocality	No	118 (77.1 %)	35549 (60.1 %)	4464 (85.7%)
	Yes	35 (22.9%)	23591 (39.9 %)	742 (14.3 %)
Extension	No	90 (56.6 %)	49129 (82.1 %)	4795 (90.4%)
	Yes	69 (43.4%)	10744 (17.9 %)	512 (9.6 %)
Radiation	None or refused	44 (27.0 %)	30701 (51.7 %)	2303 (43.6 %)
	External beam radiation therapy	27 (16.6 %)	1105 (1.9%)	163 (3.1%)
	Radioactive I-131 ablation	92 (56.4 %)	27548 (46.4 %)	2822 (53.3 %)
Surgery	Biopsy	8 (5.0 %)	1513 (2.5 %)	183 (3.4%)
	Lobectomy	14 (8.8%)	7750 (12.9 %)	1207 (22.5%)
	Subtotal or near-total thyroidectomy	8 (5.0 %)	2116 (3.5 %)	277 (5.2%)
	Total thyroidectomy	130 (81.2 %)	48771 (81.1 %)	3696 (68.9%)
Survival months	–	47.20 ± 32.69	48.99 ± 33.40	52.67 ± 34.20

ITC: insular thyroid carcinoma; CPTC: classic papillary thyroid cancer; FTC: follicular thyroid carcinoma.

### Cancer-specific and all-cause mortality rates for different histological subtypes

In our study cohort, the cancer-specific mortality rates per 1000 person-years for ITC, CPTC, and FTC were 44.68 (95% CI: 31.05–64.29), 2.51(95% CI: 2.32–2.72), and 6.68 (95% CI: 5.72–7.81), respectively (Table 2). The all-cause mortality per 1000 person-years in patients with ITC, CPTC, and FTC were 83.19(95% CI: 63.71–108.62), 10.54 (95% CI: 10.14–10.95), and 18.58 (95% CI: 16.93–20.40), respectively (Table 2).

**Table 2:** Hazard ratios of different histological types for the cancer specific deaths and all cause deaths of thyroid cancer

Histological types	Cancer-Specific Deaths,	%	Cancer-Specific Deaths per	95% CI	All Cause Deaths,	%	All Cause Deaths per	95% CI
	No.		1,000 Person-Years		No.		1,000 Person-Years	
ITC	29	17.58	44.68	31.05–64.29	55	33.33	83.19	63.71–108.62
CPTC	659	1.08	2.51	2.32–2.72	2722	4.48	10.54	10.14–10.95
FTC	178	3.28	6.68	5.72–7.81	474	8.75	18.58	16.93–20.40

ITC:insular thyroid carcinoma; CPTC: classic papillary thyroid cancer; FTC: follicular thyroid carcinoma.

### Risk factors for thyroid cancer-specific and all-cause mortality rates

According to univariate Cox regression analyses, age, sex, race, TNM stage, tumor extension, radiation, and surgical approach were significant risk factors for cancer-specific mortality. In the multivariate Cox regression model, CPTC and FTC showed no significant risk for cancer-specific mortality compared to ITC after adjusting for influential risk factors (Table 3). According to univariate Cox regression analyses, age, sex, race, TNM stage, multifocality, tumor extension, radiation, and surgical approach were found to be significant factors influencing all-cause mortality. Multivariate Cox regression analysis also revealed that ITC showed no significant risk for all-cause mortality compared to CPTC and FTC (Table 3).

**Table 3:** Risk factors for survival: outcome of thyroid cancer specific mortality and all-cause mortality

Covariate	level		Thyroid Cancer specific mortality				All cause mortality		
	Univariate Cox regression		Multivariate Cox regression		Univariate Cox regression		Multivariate Cox regression		
	Hazard Ratio (95% CI)	*p*-value	Hazard Ratio (95% CI)	*p*-value	Hazard Ratio (95% CI)	*p*-value	Hazard Ratio (95% CI)	*p*-value	
Age		1.097 (1.092–1.103)	< 0.001	1.062 (1.056-1.069)	< 0.001	1.087 (1.084–1.089)	< 0.001	1.072 (1.069–1.075)	< 0.001
Sex	Female	ref		ref		ref		ref	
	Male	2.654 (2.322–3.035)	< 0.001	1.128 (0.952-1.337)	0.164	2.456 (2.291–2.633)	< 0.001	1.594 (1.472–1.727)	< 0.001
Race	White	ref		ref		ref		ref	
	Black	1.159 (0.881–1.526)	0.292	0.927 (0.638-1.349)	0.693	1.340 (1.176–1.526)	< 0.001	1.250 (1.075–1.454)	0.004
	Other	1.409 (1.169–1.699)	< 0.001	0.983 (0.777-1.243)	0.887	0.926 (0.827–1.038)	0.188	0.797 (0.697–0.911)	0.001
histological types	ITC	ref		ref		ref		ref	
	CPTC	0.060 (0.042–0.087)	< 0.001	0.797 (0.506–1.256)	0.328	0.130 (0.100–0.170)	< 0.001	0.759 (0.553–1.043)	0.089
	FTC	0.175 (0.118–0.259)	< 0.001	1.271 (0.788–2.048)	0.325	0.238 (0.180–0.314)	< 0.001	0.918 (0.663–1.271)	0.606
T-stage T-stage	T1	ref		ref		ref		ref	
	T2	3.360 (2.385–4.735)	< 0.001	2.812 (1.944–4.067)	< 0.001	1.128 (1.006–1.264)	0.039	1.160 (1.023–1.314)	0.020
	T3	9.192 (6.991–12.085)	< 0.001	4.363 (2.950–6.453)	< 0.001	1.734 (1.579–1.903)	< 0.001	1.276 (1.091–1.493)	0.002
	T4	91.718 (71.152–118.229)	< 0.001	14.247 (9.086 22.341)	< 0.001	8.125 (7.407-–8.912)	< 0.001	2.554 (2.067–3.155)	< 0.001
N stage	N0	ref		ref		ref		ref	
	N1	4.269 (3.694–4.934)	< 0.001	2.001 (1.654–2.421)	< 0.001	1.661 (1.538–1.794)	< 0.001	1.475 (1.332–1.633)	< 0.001
M-stage	M0	ref		ref		ref		ref	
	M1	50.224 (43.821–57.564)	< 0.001	5.677 (4.618–6.978)	< 0.001	15.371 (13.996–16.881)	< 0.001	3.452 (2.980–4.000)	< 0.001
Multifocality	No	ref		ref		ref		ref	
	Yes	0.973 (0.836–1.132)	0.722	0.880 (0.741–1.045)	0.144	0.888 (0.823–0.958)	0.012	0.971 (0.893–1.056)	0.495
Extension	No	ref		ref		ref		ref	
	Yes	13.461 (11.478–15.786)	< 0.001	1.546 (1.095–2.184)	0.013	2.832 (2.631–3.049)	< 0.001	1.173 (0.985–1.396)	0.073
Radiation	None or refused	ref		ref		ref		ref	
	Radiation Beam or Rdioactive implants	16.152 (13.588–19.201)	< 0.001	2.060 (1.608–2.639)	< 0.001	4.414 (3.910–4.982)	< 0.001	1.173 (0.991–1.388)	0.063
	Radioisotopes or Radiation beam+ isotopes/implants	0.922 (0.789–1.076)	0.302	0.745 (0.610–0.910)	0.004	0.607 (0.563–0.654)	< 0.001	0.667 (0.608–0.731)	< 0.001
Surgery	Biopsy	ref		ref		ref		ref	
	Lobectomy	0.035(0.027-0.046)	<0.001	0.436(0.295-0.644)	<0.001	0.090(0.080-0.102)	<0.001	0.305(0.258-0.361)	< 0.001
	Subtotal or near-total thyroidectomy	0.082(0.060-0.113)	<0.001	0.613(0.409-0.974)	0.037	0.099(0.083-0.118)	<0.001	0.333(0.268-0.414)	< 0.001
	Total thyroidectomy	0.048(0.041-0.056)	<0.001	0.461(0.344-0.618)	<0.001	0.070(0.064-0.077)	<0.001	0.282(0.244-0.327)	< 0.001

ITC:insular thyroid carcinoma; CPTC: classic papillary thyroid cancer; FTC: follicular thyroid carcinoma;

### Adjusting for patient characteristics using PSM

**Figure 1:**
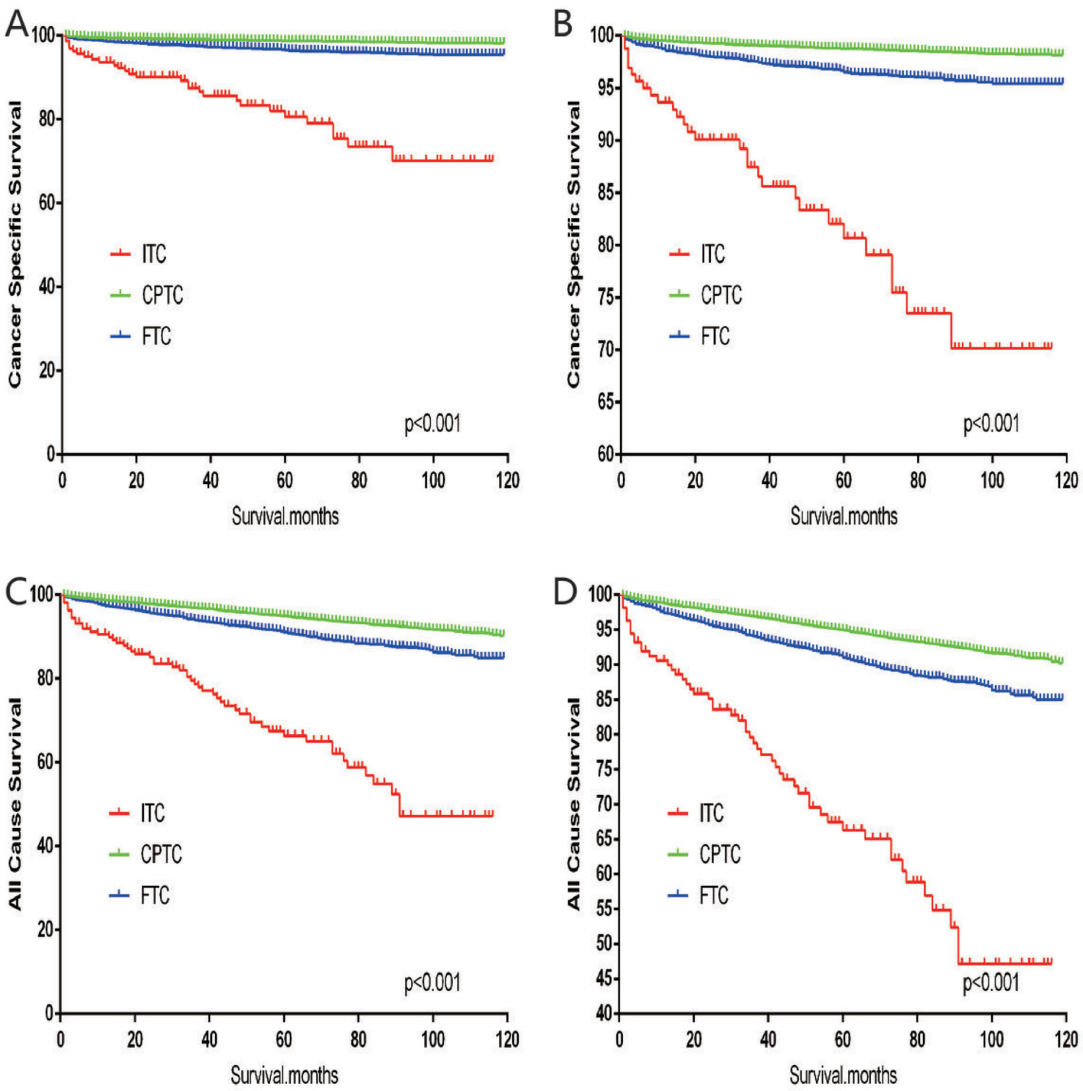
Kaplan Meier curves among patients stratified by subtype for cancer-specific mortality (A, B: Log rank test *p* < 0.0001) and all cause mortality (C, D: Log rank test *p* < 0.0001)

**Figure 2:**
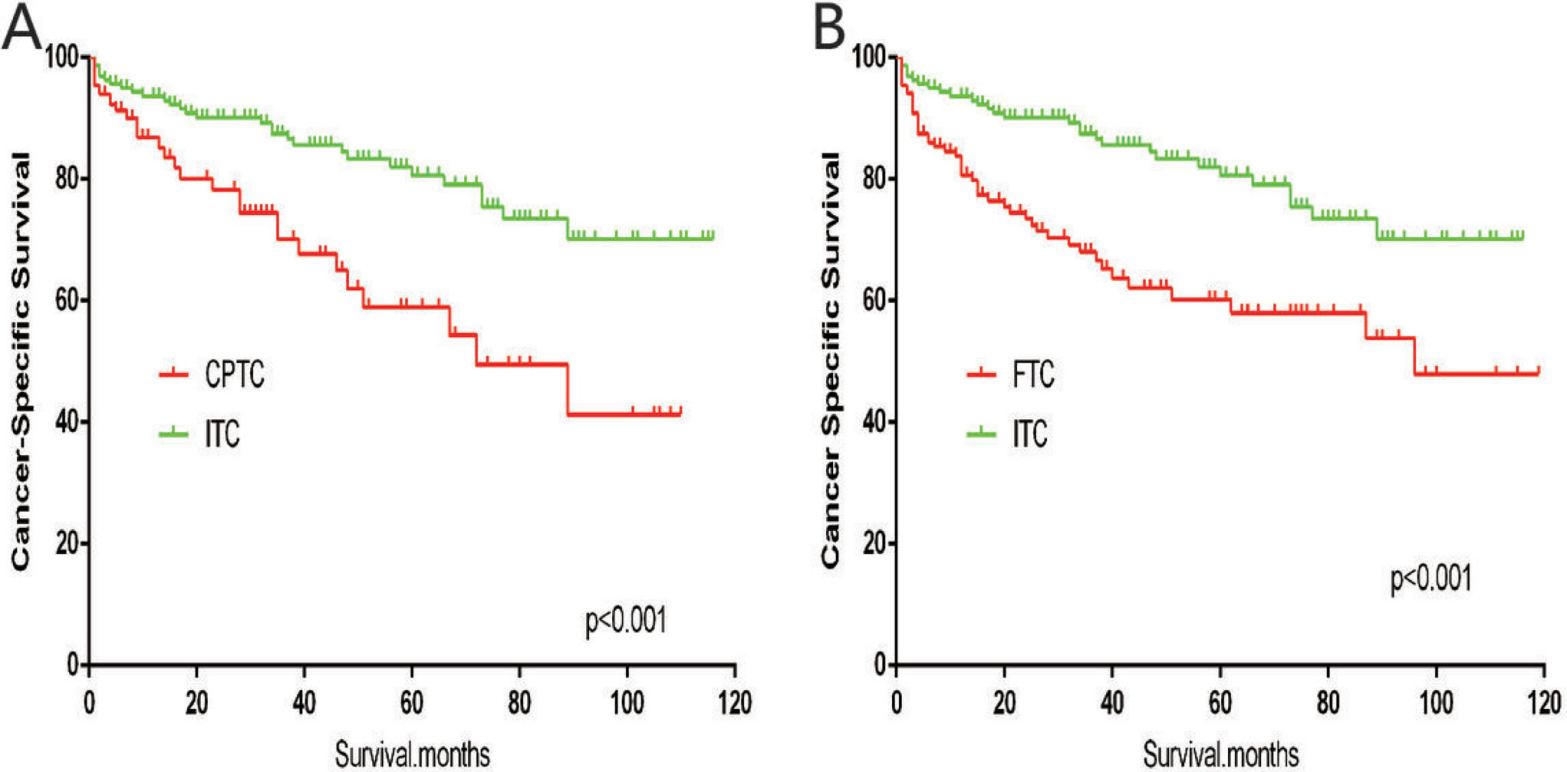
Kaplan Meier curves of cancer-specific mortality for matched subtype pairs Age, sex and race matching between ITC and CPTC (**A**), ITC and FTC (**B**).

**Figure 3:**
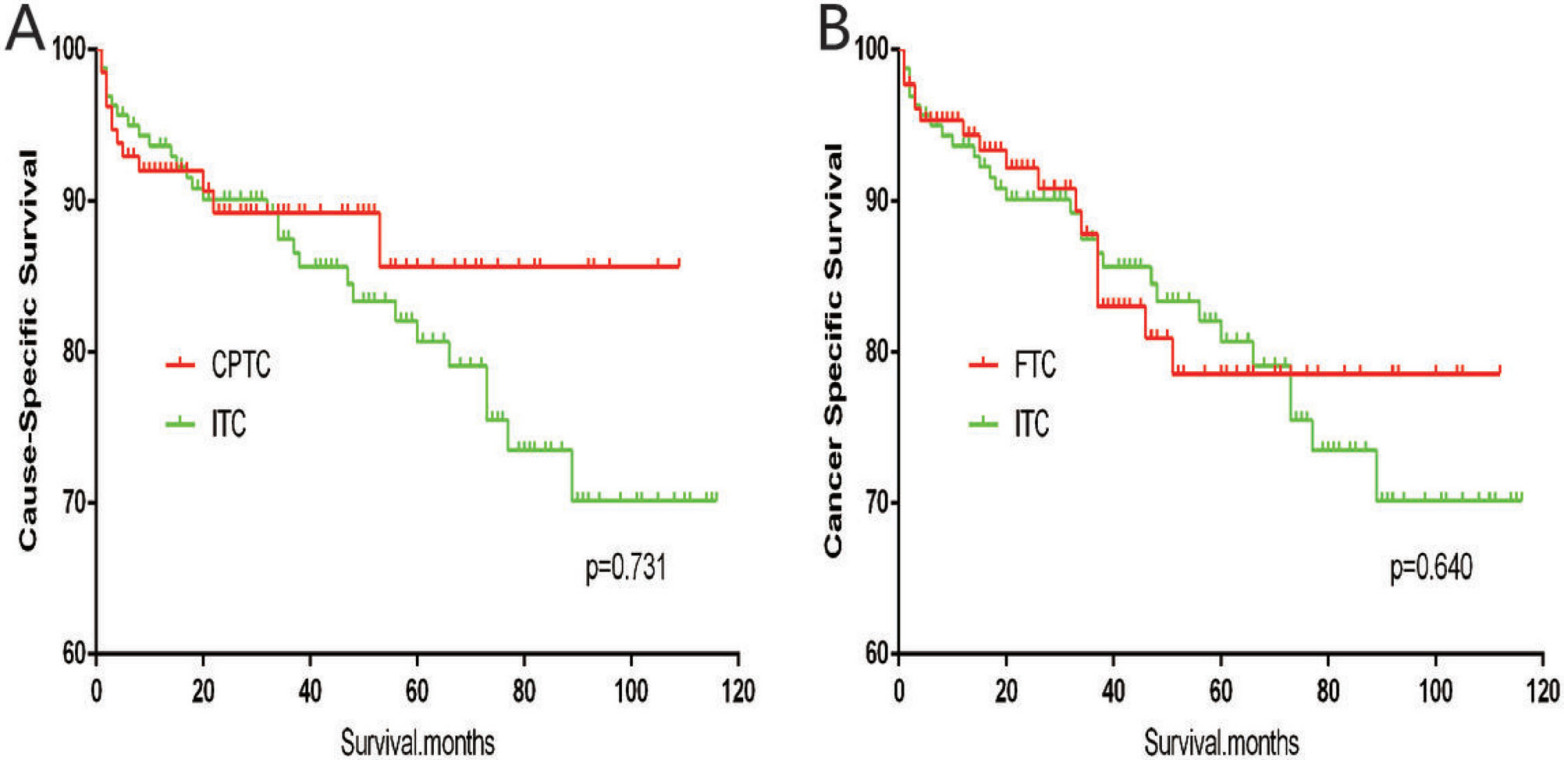
Kaplan Meier curves of cancer-specific mortality for matched subtype pairs Age, sex, race, T/N/M stage, multifocality, extension matched between ITC and CPTC (**A**), ITC and FTC (**B**).

**Figure 4:**
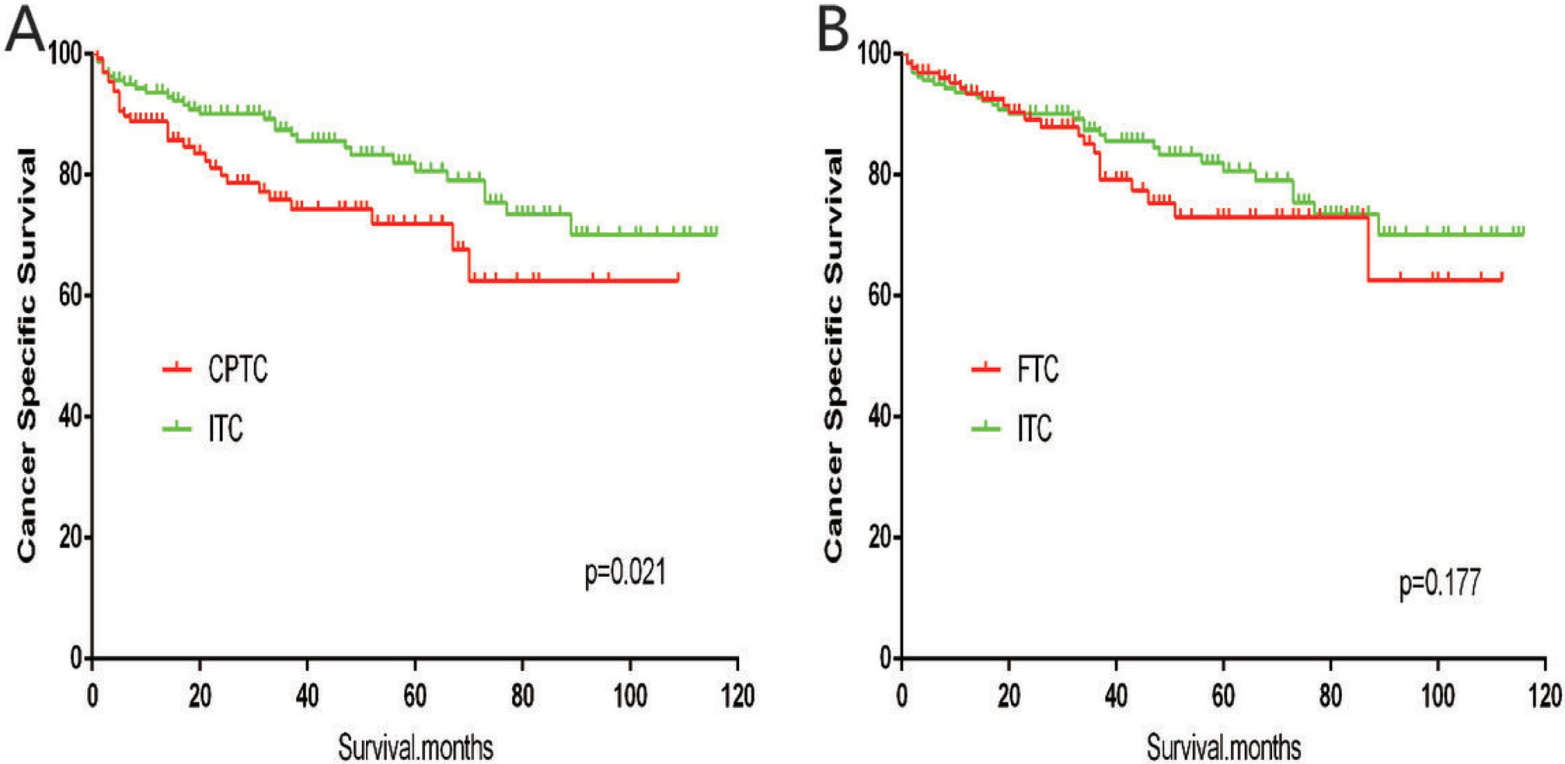
Kaplan Meier curves of cancer-specific mortality for matched subtype pairs Age, sex, race, T/N/M stage, multifocality, extension, surgery and radiation treatment matched between ITC and CPTC (**A**), ITC and FTC (**B**).

**Figure 5:**
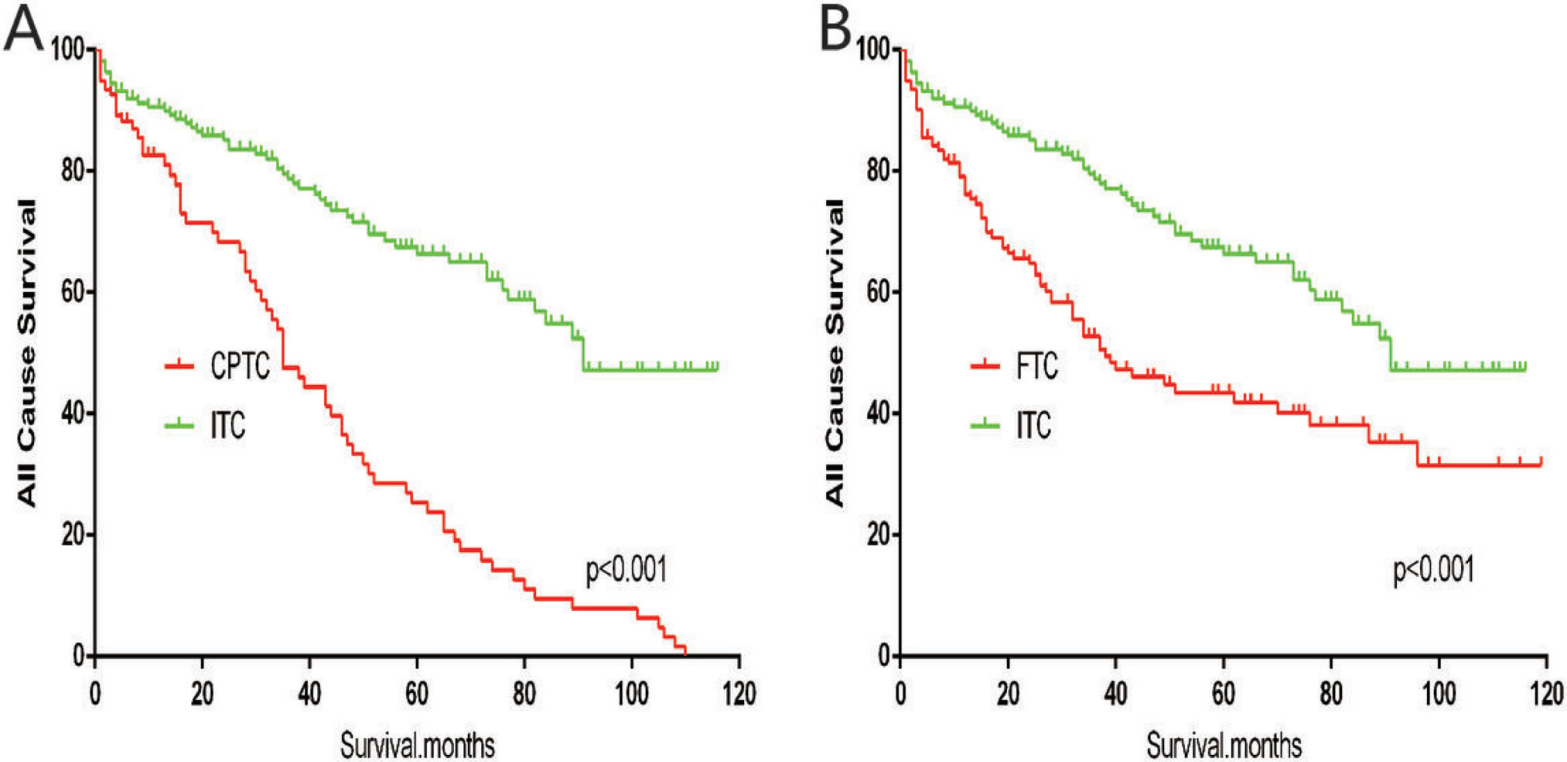
Kaplan Meier curves of all cause mortality for matched subtype pairs Age, sex and race matching between ITC and CPTC (**A**), ITC and FTC (**B**).

**Figure 6:**
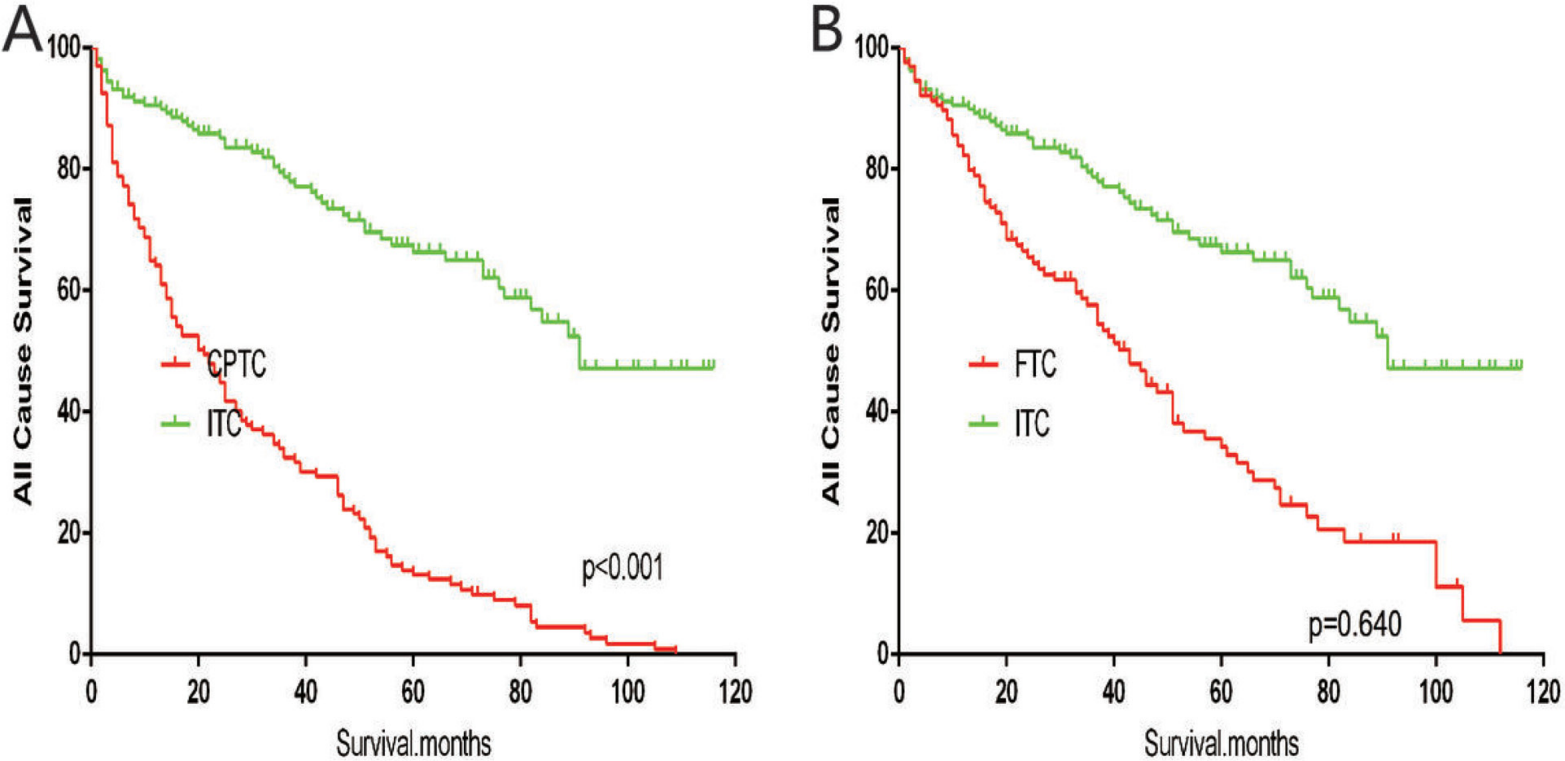
Kaplan Meier curves of all cause mortality for matched subtype pairs Age, sex, race, T/N/M stage, multifocality, extension matching between ITC and CPTC (**A**), ITC and FTC (**B**).

**Figure 7:**
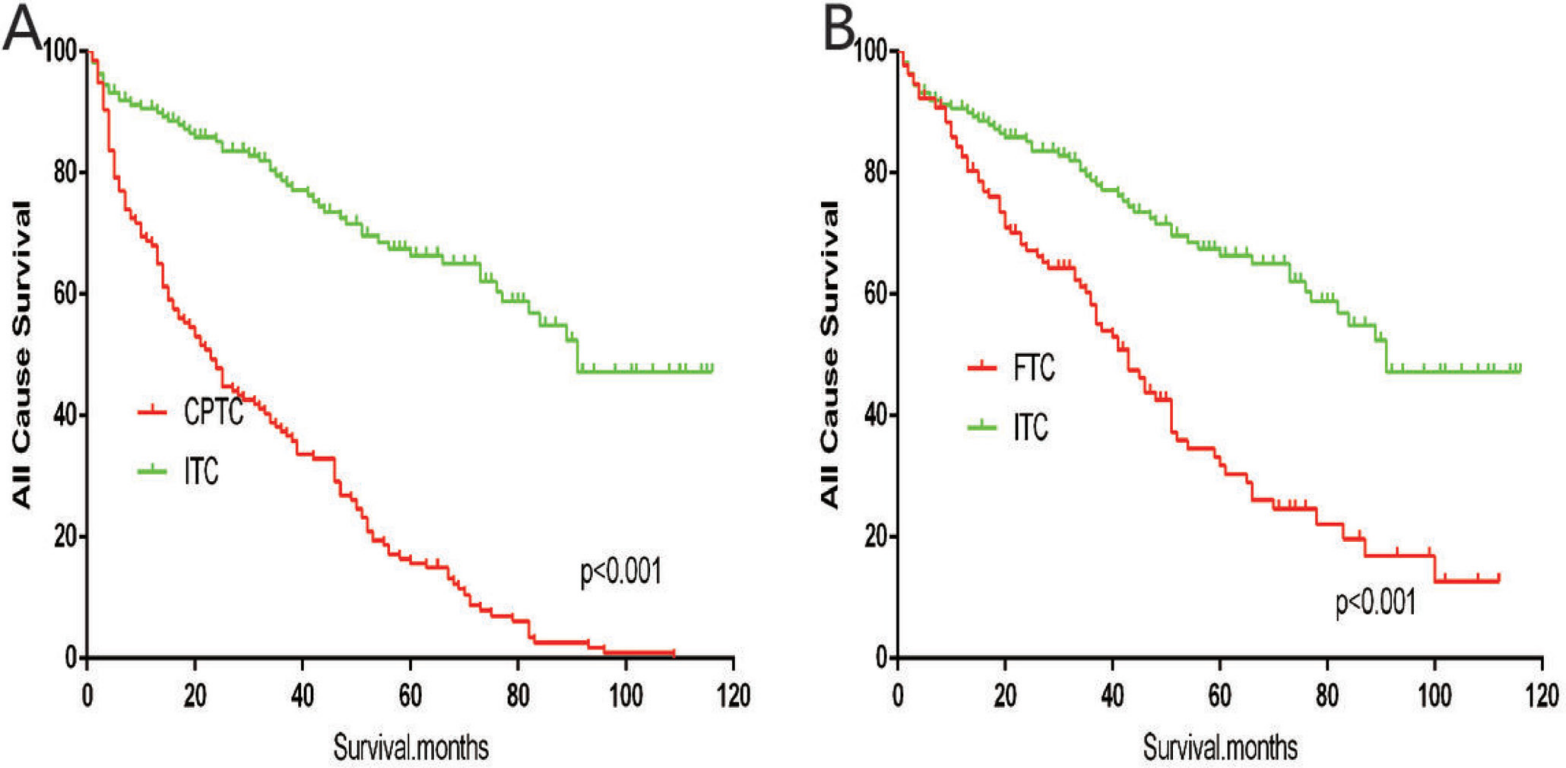
Kaplan Meier curves of all cause mortality for matched subtype pairs Age, sex, race, T/N/M stage, multifocality, extension, surgery and radiation treatment matching between ITC and CPTC (**A**), ITC and FTC (**B**).

The cancer-specific mortality rate of patients with ITC was significantly higher than that of patients with CPTC and FTC (*p* < 0.001). The all-cause mortality rate of patients with ITC was also higher than patients with CPTC and FTC (*p* < 0.001; Figure 1A–1D). To minimize selection bias, propensity scored matching analysis was performed for age, sex, race, T/N/M stage, multifocality, tumor extension, and radiotherapy approaches. On survival analysis, patients with ITC had a better prognosis for cancer-specific survival compared to patients with CPTC and FTC after PSM for age, sex, and race (*p* < 0.001 for both; Figure 2A–2B). Furthermore, there were no significant differences in cancer-specific mortality between ITC and CPTC patients after PSM for age, sex, race, T/N/M stage, multifocality, and tumor extension (*p* = 0.731; Figure 3A). Patients with ITC were observed to have cancer-specific survival rates similar to patients with FTC (*p* = 0.640; Figure 3B). After matching for all potential influencing factors (including radiotherapy), the prognosis for patients with ITC was similar to that for patients with FTC, but better than that for patients with CPTC (*p* = 0.177 and *p* = 0.021, respectively; Figure 4A–B). According to survival analysis for all-cause mortality, ITC had a better prognosis compared to CPTC and FTC after matching for age, sex, and race (all *p* < 0.001; Figure 5A–5B). Similar results were obtained after matching for age, sex, race, T/N/M stage, multifocality, and tumor extension between CPTC and ITC (*p* < 0.001; Figure 6A). However, the prognosis of patients with ITC showed no statistical difference compared to those with FTC (*p* = 0.640; Figure 6B). After matching for all influential factors including radiation treatment, patients with CPTC and FTC showed poorer all-cause mortality rates compared to patients with ITC (both *p* < 0.001; Figure 7A–7B).

## DISCUSSION

ITC is a distinctive and rare thyroid cancer that is characterized by nests or “insulae” of small uniform neoplastic cells, and has a propensity to metastasize to regional lymph nodes and distant sites [11]. ITC is considered morphologically and biologically intermediate compared to well-differentiated thyroid carcinomas (PTC and FTC) on one hand and anaplastic carcinoma of the thyroid on the other; it was incorporated into the World Health Organization classification of poorly differentiated thyroid carcinoma in 2004 [21, 22]. According to the literature, disease-related mortality due to ITC is higher than that due to DTC [15, 23–25], and its insular histotype is independently associated with patient survival [26, 27]. Because of sample size limitations owing to the overall rarity of ITC, the paucity of available studies have precluded the accurate determination of this disease's prognosis.

In our analysis of the SEER data, histological subtype was not an independent risk factor for cancer-specific or all-cause mortality. Before performing PSM, ITC was observed to have a poorer prognosis than CPTC and FTC; however, after adjusting for all potentially influencing factors including demographic data and radiation treatment, the cancer-specific mortality rate of patients with ITC was similar to that of patients with FTC and better than that of patients with CPTC. Likewise, the all-cause mortality rate in patients with ITC was better than those in patients with CPTC and FTC. This result was unexpected and maybe have potential prognostic and therapeutic implications for patients with ITC , as it was inconsistent with previously published data [15, 24, 25, 28, 29] ,and it maybe more trustworthy because we performed PSM analyses to adjust for biases from different baseline characteristics of two histotype groups , Surgeons have not yet established the best treatment option for ITC, and our results may provide guidance for clinicians and may also influence the new American Thyroid Association guidelines.

Patients with ITC have reportedly been recommended for total thyroidectomy, lymph node dissection, and radioactive iodine owing to a high incidence of nodal involvement as well as their poor prognoses [15, 30]. However, in our current study, the prognosis of patients with ITC was better than that of CPTC and FTC after matching for all potentially influencing factors, and only 24% of patients with ITC experienced lymph node metastasis. Therefore, patients with ITC may not require aggressive treatment unless certain risk factors such as multifocality, tumor extension, and lymph node metastasis (as detected by preoperative ultrasonography) exist.

Previous molecular studies have exposed the roles of various oncogenes in different types of thyroid tumors [31, 32]. The discovery of novel molecular-based prognostic markers for thyroid cancer, such as *RET-PTC*, *RAS*, *BRAF* (V600E), *PTEN* and *TERT* mutations, are among the most notable developments in thyroid cancer-related medicine [33]. However, the role of molecular mechanisms and genetic events in determining the growth and biologic aggressiveness of ITC remains to be explained. Pilotti et al. [16] reported *RAS* gene family point mutations involving a high proportion of CAA→AAA transversions at codon 61 of the *N-RAS* gene in ITC; however, the same abnormality was found with similar frequency in invasive follicular thyroid carcinoma [34]. Likewise, mutations in the *p53* gene are also nonspecific, as they have been described in both patients with ITC and in those with anaplastic carcinoma [23, 35]. The further exploration of the molecular changes in ITC may be helpful for tailoring therapeutic interventions, and patient enrollment in clinical trials should be encouraged when systemic therapy for disseminated disease is required.

Our study had several limitations. First, our dataset lacked information on recurrence, which may have led to the overestimation of cancer-specific and all-cause mortality rates. Another limitation is that family history, vascular invasion, and other histologic findings were not evaluated or considered in our study. Furthermore, the SEER is a United States database, and the findings may not be applicable worldwide.

In summary, we found that patients diagnosed with ITC had similar prognoses as those with FTC and better prognoses than those with CPTC; this was contrary to the findings of previous (albeit smaller-sized) studies. Our findings ought to serve as a reference for the planning of future therapies by adjusting the intensity of the interventions based on the prognosis of this disease being more favorable than previously thought.

## MATERIALS AND METHODS

### Ethics statement and study population

This investigation was conducted in accordance with the ethical standards set by the Declaration of Helsinki, and pursuant to national and international guidelines. The study was approved by Union Hospital's institutional review board.

We used the National Cancer Institute's SEER database to investigate patients with thyroid cancer, including CPTC, ITC, and FTC. The SEER project began in 1973; it is the only comprehensive source of population-based cancer information in the United States, and is supported by both the Centers for Disease Control and Prevention and the National Cancer Institute. This database reports cancer-related data that include the incidence, prevalence, mortality, population-based variables, and primary tumor characteristics (i.e., histological subtype) from multiple geographic regions within the United States.

### Data collection and analysis

Patients diagnosed with CPTC, ITC, and FTC between 2004 and 2013 were identified using the SEER database and combinations of the International Classification of Diseases for Oncology site code C73.9 (i.e., thyroid, papillary, and/or follicular histology). The diagnosis codes included in the study were: “papillary carcinoma”, “papillary adenocarcinoma”, “follicular carcinoma”, ”papillary carcinoma, insular”, “follicular adenocarcinoma”, and “papillary and follicular adenocarcinoma”. Thyroid cancer-specific and all-cause survival rates were compared among 66323 patients with CPTC, ITC, and FTC. Age, sex, race, T/N/M stage, multifocality, tumor extension, and radiotherapy (i.e., none or refused, external beam radiation therapy, and radioactive I-131 ablation) were evaluated in patients with different histological subtypes.

### Statistical analyses

Patient survival curves depicting thyroid cancer-specific and all-cause mortality were calculated by using Kaplan-Meier analyses; the log-rank test was used to determine statistical differences in survival. PSM analysis was conducted to control for potential baseline confounding factors. The propensity score analysis generally calculated using logistic regression with group as dependent, covariates as independent variables [36, 37]. Cox proportional hazards regression analyses were performed to estimate hazard ratios with 95% CIs and to show the magnitude of the effect of different histological subtypes on cancer-specific and all-cause mortality rates. All *p*-values were 2-sided, and *p*-values < 0.05 were considered statistically significant throughout the study. Analyses were performed using the SPSS software version 23.0, Stata/SE version 12 (Stata Corp.), and GraphPad Prism version 6 (GraphPad Software Inc.).

## Abbreviations

CI: confidence interval
CPTC: classic papillary thyroid cancer
DTC: differentiated thyroid cancer
FTC: follicular thyroid cancer
ITC: insular thyroid carcinoma
PSM: propensity score matching
PTC: papillary thyroid cancer
SEER: Surveillance, Epidemiology, and End Results
